# A multicentered retrospective cohort study comparing JAK inhibitor therapies in moderate-to-severe ulcerative colitis

**DOI:** 10.1093/crocol/otag045

**Published:** 2026-05-26

**Authors:** Aditi Kumar, Joshua Baxter, Peter Rimmer, Ben Noble, Joshua Bower, Mujeeb Ullah Makki, Ayiesha Hatta, Anmol Chikhlia, Jonathan Cheesbrough, Ben Disney, Josh Muir, Mariya Karova, Jeffrey Butterworth, Nidhi Sagar, Ismaeel Al-Talib, Jasbir Nahal, Nasima Ali, Vandana Sagar, Fumi Varyani, Sam Smith, Stephanie Bourne, Yu-Kai Hsu, Almoiez Eltahir, Shanika de Silva, Philip Harvey

**Affiliations:** Department of Gastroenterology, The Royal Wolverhampton NHS Trust, Wolverhampton, UK; Department of Gastroenterology, Walsall Manor NHS Trust, Walsall, UK; Department of Gastroenterology, Queen Elizabeth Hospital Birmingham, Birmingham, UK; Department of Gastroenterology, The Royal Wolverhampton NHS Trust, Wolverhampton, UK; Department of Gastroenterology, University Hospitals Coventry & Warwickshire, Coventry, UK; Department of Gastroenterology, University Hospitals Coventry & Warwickshire, Coventry, UK; Department of Gastroenterology, University Hospitals Coventry & Warwickshire, Coventry, UK; Department of Gastroenterology, University Hospitals Coventry & Warwickshire, Coventry, UK; Department of Gastroenterology, Walsall Manor NHS Trust, Walsall, UK; Department of Gastroenterology, University Hospitals Coventry & Warwickshire, Coventry, UK; Department of Gastroenterology, Shrewsbury & Telford NHS Trust, Shrewsbury, UK; Department of Gastroenterology, Shrewsbury & Telford NHS Trust, Shrewsbury, UK; Department of Gastroenterology, Shrewsbury & Telford NHS Trust, Shrewsbury, UK; Department of Gastroenterology, Walsall Manor NHS Trust, Walsall, UK; Department of Gastroenterology, Walsall Manor NHS Trust, Walsall, UK; Department of Gastroenterology, Walsall Manor NHS Trust, Walsall, UK; Department of Gastroenterology, Sandwell General Hospital, Birmingham, UK; Department of Gastroenterology, Sandwell General Hospital, Birmingham, UK; Department of Gastroenterology, Sandwell General Hospital, Birmingham, UK; Department of Gastroenterology, Worcester Royal Hospital, Worcester, UK; Department of Gastroenterology, Royal Stoke University Hospital, Stoke, UK; Department of Gastroenterology, Dudley Group NHS Trust, Dudley, UK; Department of Gastroenterology, Dudley Group NHS Trust, Dudley, UK; Department of Gastroenterology, Dudley Group NHS Trust, Dudley, UK; Department of Gastroenterology, The Royal Wolverhampton NHS Trust, Wolverhampton, UK

**Keywords:** ulcerative colitis, JAK inhibitors, upadacitinib, filgotinib, tofacitinib, inflammatory bowel disease

## Abstract

**Background:**

Tofacitinib, filgotinib, and upadacitinib are Janus kinase inhibitors (JAKi) that have demonstrated efficacy in ulcerative colitis (UC) against placebo. This study aims to compare the clinical efficacy between these drugs.

**Methods:**

This is a multicentered, retrospective cohort study. Patients with UC starting a JAKi were recruited between 2018 and 2024 when starting their first JAKi. Clinical remission and response, based on clinical scores, calprotectin, and endoscopic measurement, were assessed at 3 and 6 months. Both independent and combined variable outcomes were analyzed.

**Results:**

Two hundred and seventy patients were included in the analysis, of which 70 (26%) were on upadacitinib, 51 (19%) on filgotinib, and 149 (55%) on tofacitinib. Clinical, biochemical, and endoscopic remission at 6 months was 91%, 71%, and 80% for upadacitinib; 78%, 67%, and 50% for filgotinib; 73%, 51%, and 44% for tofacitinib. Upadacitinib demonstrated significantly greater clinical response (*P* = .027) and remission (*P* = .037) rates at 6 months than tofacitinib. Drug persistence at 12 months was 86% for upadacitinib, 72% for filgotinib, and 69% for tofacitinib. Upadacitinib demonstrated significantly greater 6-month remission rates compared to tofacitinib in the bio-exposed cohort (71% vs 52%, *P* = .022) and in the bio-naïve cohort (93% vs 50%, *P* = .009). The incidence rates for hospital admissions were 10.2, 21.9, and 14.1 and for colectomies were 6.7, 10.0, and 5.0 per 1000 patient-months at risk for upadacitinib, filgotinib and tofacitinib, respectively.

**Conclusion:**

This study demonstrates that upadacitinib is more likely to achieve 6-month response and remission compared to tofacitinib. In both bio-naïve and bio-exposed patients, upadacitinib was more likely to achieve remission at 6 months. The efficacy of JAKi does not appear diminished by prior biologic use.

## Teaser

This multicentered retrospective cohort study compares efficacy between the 3 Janus kinase inhibitors in moderate-to-severe ulcerative colitis. The results demonstrate that upadacitinib is more likely to achieve 6-month response and remission. In both bio-naïve and bio-exposed patients, upadacitinib was more likely to achieve remission at 6 months.

Key Messages **What is already known?** 

- Tofacitinib, filgotinib and upadacitinib are Janus kinase inhibitors (JAK) inhibitors, which have shown strong efficacy against placebo for patients with mild to moderate ulcerative colitis
- There is a scarcity of head-to-head JAK inhibitor studies.

**What is new here?** 

- Upadacitinib demonstrated significantly greater clinical response and remission rates at 6 months than tofacitinib.
- Upadacitinib demonstrated significantly greater 6-month remission rates compared to tofacitinib in the bio-exposed cohort and in the bio-naïve cohort.

**How can this study help patient care?** 

- This study demonstrates upadacitinib to have greater efficacy in ulcerative colitis and its benefit was not diminished in the bio-exposed cohort, making it a useful option for treatment resistant patients.

## Introduction

Ulcerative colitis (UC) is a chronic relapsing remitting disease affecting the gastrointestinal tract. In the last two decades, there have been significant improvements in both the understanding of the disease process and the identification of novel therapeutic targets.

The Janus kinase inhibitors (JAKi) are targeted synthetic drugs with low molecular weight. They are the first small molecule drug modality to be licensed in IBD with an oral route of administration. Tofacitinib is the first JAKi licensed for treatment of moderate to severe UC in the UK in 2018, followed by filgotinib in 2022 and Upadacitinib^1^ in 2023. Upadacitinib is the first and only JAKi that is licensed for both UC and Crohn’s disease. In contrast to anti-TNF inhibitors that block a single cytokine of a few specific molecules, JAKi can block multiple cytokines from different inflammatory pathways simultaneously, thereby potentially improving the therapeutic response.^2^ They are attractive to both healthcare professionals and patients due to their quick absorption and rapid onset of action, short half-life and lack of immunogenicity.^3^ They also have the added advantage of reducing significant healthcare and environmental costs that are associated with the delivery and administration of intravenous and subcutaneous medications.^4^ Furthermore, small molecules have more predictable and stable pharmacokinetics, such that additional storage costs are not needed, making them much simpler and less expensive to manufacture than biologic medications.^5^ These drugs can be particularly useful in the case of concurrent infections or pre-operatively, when rapid drug elimination is advantageous.

While all three JAKi medications have demonstrated strong efficacy against placebo from their individualized randomized controlled trials,^6–8^ there has been a scarcity of head-to-head studies between the three drugs. This real-world cohort study compares the clinical efficacy for induction of remission between the three licensed JAKi drugs in patients with UC.

## Methods

### Study design

This was a retrospective cohort study from multiple centers in the UK. Data were collected from January 2018 to June 2024. Patients with UC were included in the study during this time period if they were initiated on their first JAKi, irrespective of previous advanced therapies. The primary outcome of this study was to compare response and remission rates between the three JAKi medications at 3 and 6 months. The secondary outcomes included assessing steroid-free remission at 3 and 6 months, exploring significant adverse effects, colectomy and hospitalization rates. Data were collected at 3 and 6 months, but investigations that were completed within 4 weeks of the timepoint were accepted.

Patient inclusion criteria for the analysis included:

- Patients with a diagnosis of UC
- Treatment with JAKi was initiated between January 2018 to June 2024.
- the first JAKi used by that patient.
- Sufficient data were available to calculate clinical assessment scores, fecal calprotectin (FCP) and/or endoscopic outcomes to demonstrate that the 3-month remission outcome was achieved.

Exclusion criteria:

- Patients under the age of 16 years.
- Crohn’s disease
- Indeterminate colitis
- Previous JAKi use

### Definitions

Treatment response and remission were evaluated using clinical, biochemical, and/or endoscopic measures. Clinical assessment was determined from the partial Mayo score (stool frequency, rectal bleeding and physician global assessment) or the simple clinical colitis activity index (SCCAI). Biochemical assessment was measured using FCP. Endoscopic assessment was measured using either the Mayo endoscopic score (MES), partial mayo (pMayo), or the ulcerative colitis endoscopic index of severity (UCEIS).

Response to treatment was considered to be met if patients had 50% reduction in their clinical pMayo score or SCCAI, 50% reduction in FCP from baseline, a reduction in either MES, pMayo or UCEIS by 3 points or more, or sustained score of <3. Disease remission was defined as clinical pMayo score <2 or SCCAI ≤ 2, FCP < 250, MES ≤ 1 or pMayo ≤ 2, UCEIS ≤ 1, or SCCAI score ≤ 2. Response and remission results by each metric at 3 and 6 months are reported individually.

Different clinical teams utilize personalized strategies for assessing response to treatment. For example, some may assess response by endoscopy, while others may simply use FCP trends. Therefore, a combined outcome measure was reported that integrated available clinical data based on the above thresholds. If multiple data points were available, such as a clinical score and an endoscopy score, a hierarchy was applied for outcome measures. Endoscopic scores superseded FCP which superseded clinical activity scores, and this is in keeping with accepted clinical practice. Unadjusted outcomes are reported for response and remission at 3 and 6 months. Multivariable analyses were also undertaken using logistic regression to correct for baseline factors.

If remission or response was demonstrated at 3 months with no further data available at 6 months, then it was presumed the patient remained in this state at 6 months. If a patient stopped taking their JAKi, they were considered to have failed both response and remission.

Persistence on JAKi and adverse events were collected for the duration of JAKi use, or until the point of data collection.

### Statistical analysis

Baseline demographics including age, gender, smoking history, prior biologic use and disease extent are reported as *n* (%) for each JAKi and the total cohort. Remission and response outcome measures are reported as *n* (%). The proportion of patients meeting remission criteria at baseline, 3 months, and 6 months are reported separately for each of clinical scores, calprotectin, and endoscopic measurements.

Statistical analysis was performed using STATA version 15. Statistical significance between each drug was assessed using a Chi^2^ test for individual and combined response and remission outcomes at 3 and 6 months. Baseline variables were assessed for statistical significance with Chi^2^ or Kruskal-Wallis tests. *P* value of <.05 were considered to be statistically significant. Persistence was described using the Kaplan-Meier survival analysis. Logistic regression models were constructed for combined outcomes of response and remission at 3 and 6 months. Goodness of fit was assessed using a Hosmer-Lemeshow test.

## Results

### Patient demographics

A total of 270 patients were included in the analysis. Seventy patients (26%) were on upadacitinib, 51 (19%) on filgotinib, and 149 (55%) on tofacitinib. The majority of patients were male (*n* = 154, 57%), nonsmokers (*n* = 206, 76%), had either distal (*n* = 113, 42%) or pancolitis (*n* = 128, 47%), and had been on at least 1 previous biologic (*n* = 217, 80%) at the time of JAKi initiation. Eighty-seven percent (129/149) of patients on tofacitinib had exposure to a previous biologic compared to 80% (56/70) for upadacitinib and 63% (32/51) for filgotinib. Corticosteroids were given at the time of starting the JAKi in 52%, 56% and 59% (*P* = .672) of patients who received upadacitinib, filgotinib, and tofacitinib, respectively. Following induction, only 3 patients on filgotinib received 100 mg once daily dosing and 5 patients on upadacitinib received 15 mg once daily for maintenance. The remaining patients received the standard maintenance regime. Table 1 provides the full patient demographic breakdown and baseline disease characteristics.

**Table 1:** Patient demographics at the time of JAKi initiation.

	Tofacitinib	Filgotinib	Upadacitinib	*P*-value
**Number**	149 (55%)	51 (19%)	70 (26%)	
**Median age range**	36-40	41-45	36-40	.651
**Gender**				
** Female**	73 (49%)	17 (33%)	26 (37%)	.090
** Male**	76 (51%)	34 67%)	44 63%)	
**Smoking status**				
** Current smoker**	1 (0.7%)	3 (6%)	1 (1%)	.413
** Never smoked**	110 (74%)	39 (76%)	57 (81%)	
** Ex-smoker**	21 (14%)	8 (16%)	6 (9%)	
** Unknown**	17 (11%)	1 (2%)	6 (9%)	
**Disease extent**				
** Proctitis (E1)**	12 (24%)	2 (4%)	7 (10%)	.154
** Left-sided disease (E2)**	64 (43%)	15 (29%)	34 (49%)	
** Pancolitis (E3)**	72 (48%)	30 (59%)	26 (37%)	
** Unknown**	1 (0.7%)	4 (8%)	3 (4%)	
**Previous biologic use**				
** No prior biologic**	20 (13%)	29 (37%)	14 (20%)	.001
** 1 previous biologic**	67 (45%)	14 (27%)	28 (40%)	
** 2 biologics**	51 (34%)	14 (37%)	20 (29%)	
** 3 or more biologics**	11 (7%)	4 (8%)	8 (11%)	
**Baseline corticosteroid**				
** Received**	85 (59%)	28 (56%)	36 (52%)	.606
** Baseline disease activity**				
** Partial Mayo score** ^a^	6 (4-7.5)	5 (4-7)	6 (4-8)	.526
** SCCAI** ^a^	9 (6-11)	9 (6-10)	6 (5-10)	.248
** Fecal calprotectin** ^a^	1408 (600-1800)	1531 (876-1800)	1020 (400-1800)	.152
** Mayo endoscopic score** ^a^	2 (2-3)	2 (2-3)	3 (2-3)	.385
** UCEIS** ^a^	6 (5-7)	4 (2-7)	5 (4.5-6.5)	.331

Disease extent was described using the Montreal classification.

Abbreviations: SCCAI, simple clinical colitis activity index; UCEIS, ulcerative colitis endoscopic index of severity.

a Reported as median (interquartile range).

No statistically significant differences were noted between baseline disease and demographic variables with the exception of prior biologic use. Filgotinib had a higher proportion of bio-naive patients than either tofacitinib or upadacitinib (*P* = .001).

### Response and remission outcomes individually by clinical, biochemical, and endoscopic score at 3 and 6 months

**Figure 1:**
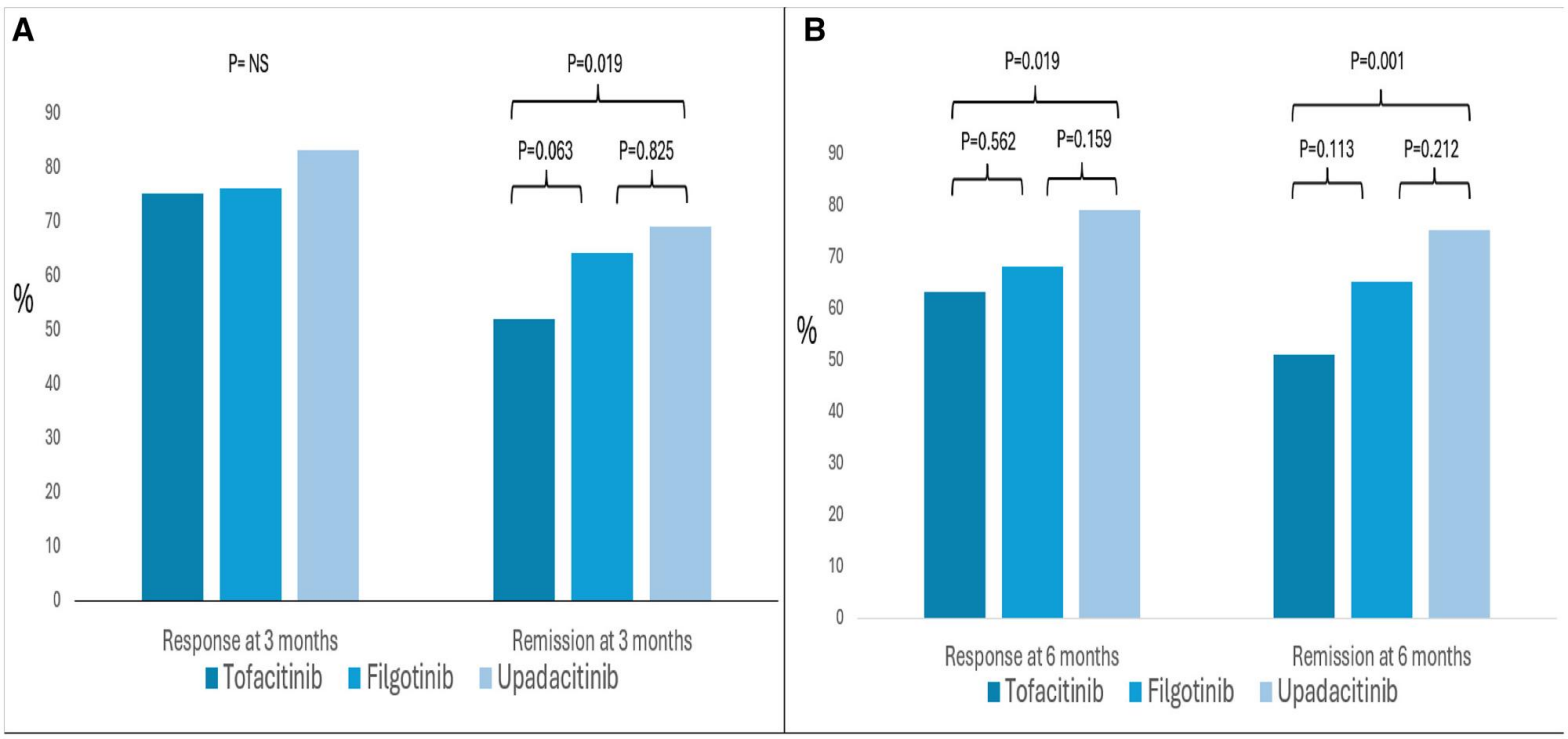
(A) The proportion of patients using tofacitinib, filgotinib, and upadacitinib meeting response and remission outcomes at 3 months. (B) The proportion of patients using tofacitinib, filgotinib and upadacitinib meeting response and remission outcomes at 6 months.

At 3 months, patients on upadacitinib demonstrated clinical remission in 74% (34/46), biochemical remission (FCP < 250) in 69% (27/39) and endoscopic remission in 83% (5/6). Clinical response was observed in 87% (34/39) and biochemical response in 86% (36/42). At 6 months, this improved to 91% (21/23) in clinical remission, 71% (17/24) in biochemical remission and 80% in endoscopic remission (4/5) (Figure 1). Clinical response was observed in 100% (23/23) and biochemical response in 84% (21/25).

Patients on filgotinib at 3 months demonstrated clinical remission in 84% (26/31), biochemical remission in 71% (17/24), and endoscopic remission in 67% (2/3). Clinical response was observed in 89% (25/28), and biochemical response in 92% (24/26). At 6 months, this reduced to 78% (14/18) in clinical remission, 67% (10/15) in biochemical remission, and 50% (1/2) in endoscopic remission. Clinical response was observed in 84% (16/19), and biochemical response in 76% (13/17).

At 3 months, patients on tofacitinib demonstrated clinical remission in 66% (73/111), biochemical remission in 51% (41/80) and endoscopic remission in 25% (3/12). Clinical response was observed in 87% (34/39) and biochemical response in 86% (36/42). At 6 months, 73% (59/81) were in clinical remission, 51% (37/68) remained in biochemical remission, and 44% (4/9) had endoscopic remission. Clinical response was observed in 82% (63/77), and biochemical response in 75% (55/73).

At 6 months, upadacitinib demonstrated significantly greater clinical response (100% vs 87%, *P* = .027) and remission rates (91% vs 73%, *P* = .037) than tofacitinib. Filgotinib was also shown to have significantly greater clinical remission rates at 3 months compared to tofacitinib (84% vs 66%, *P* = .033). Full results are shown graphically in Figure 1 and in Appendix Table S1.

### Persistence

**Figure 2:**
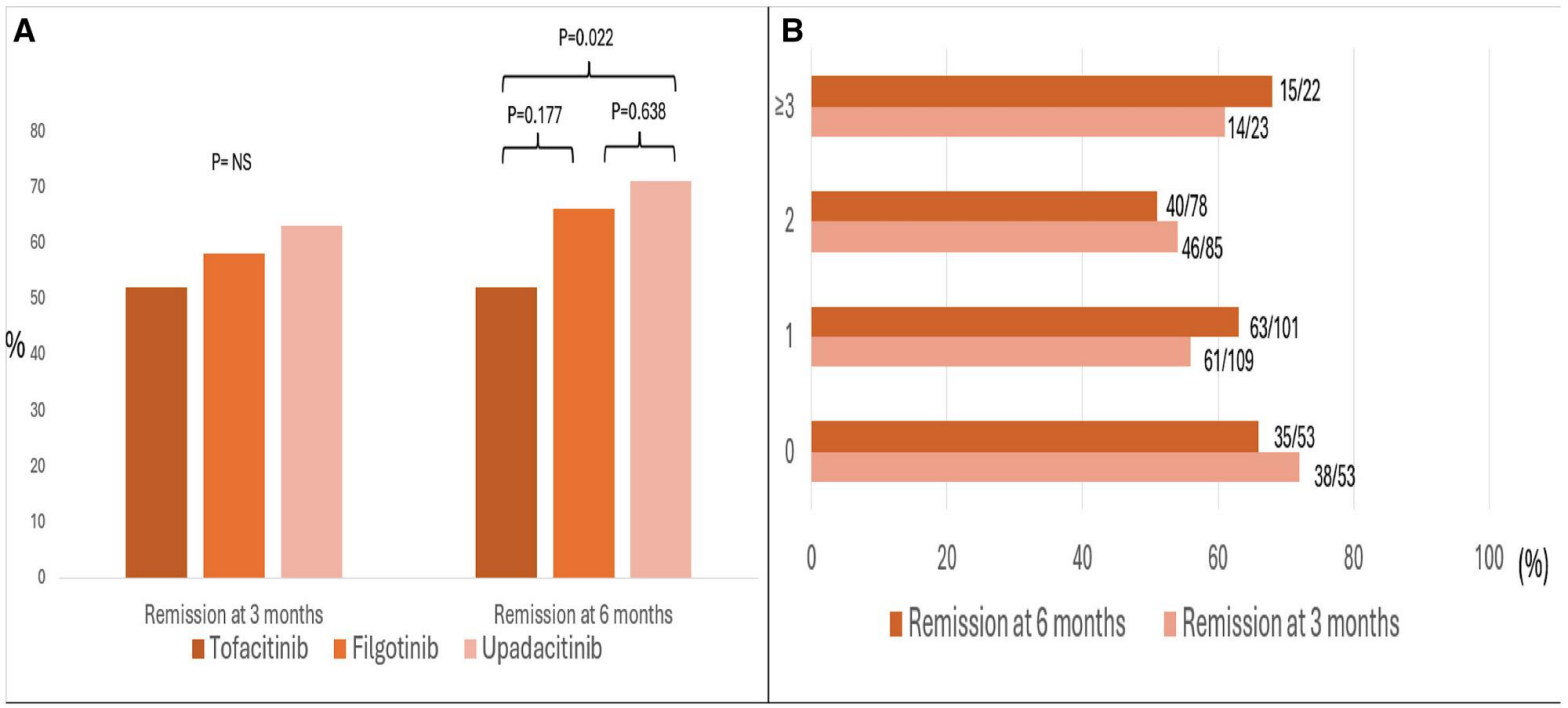
(A) The proportion of bio-exposed patients using tofacitinib, filgotinib and upadacitinib meeting remission outcome at 3 and 6 months. (B) The proportion of bio-exposed patients using any JAK inhibitor meeting remission outcome at 3 and 6 months.

The persistence rates at 12 months were 86% for upadacitinib, 72% for filgotinib, and 69% for tofacitinib (Figure 2).

### Combined response and remission rates

**Figure 3:**
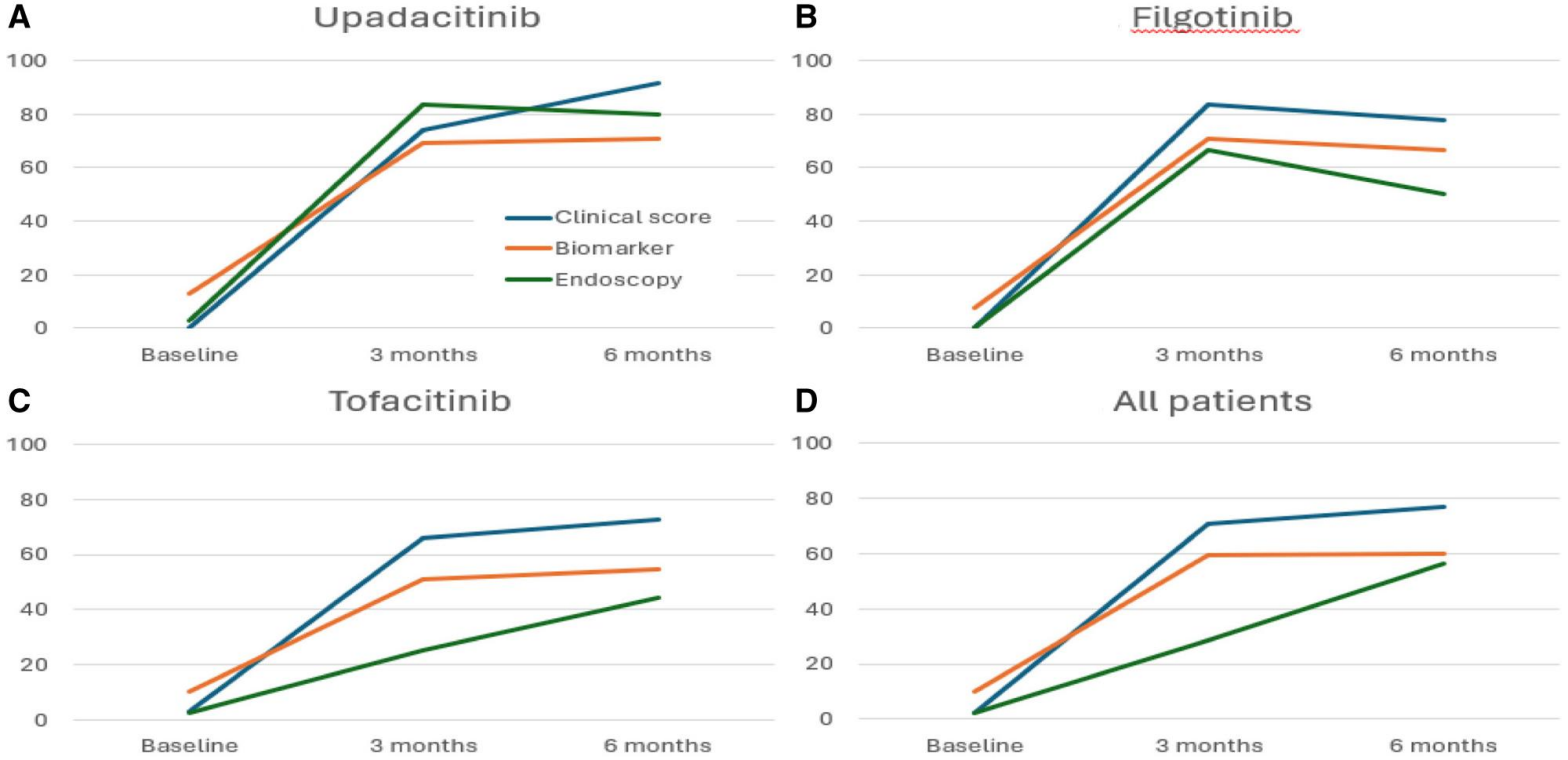
(A–D) Proportion of patients meeting remission criteria by clinical score, fecal calprotectin biomarker, and endoscopic assessment at baseline, 3 months, and 6 months for all JAKi and each drug individually.

At 3 months combined response to upadacitinib, filgotinib, and tofacitinib was demonstrated in 83% (55/66), 76% (39/51), and 75% (109/146) of patients, respectively. At 3 months combined remission was demonstrated in 69% (48/70), 64% (34/51), and 52% (77/149), respectively (see Figure 3).

At 6 months, combined response to upadacitinib, filgotinib, and tofacitinib was demonstrated in 79% (54/68), 68% (34/50), and 63% (92/145), respectively. Combined remission was achieved in 75% (49/65), 65% (31/48), and 51% (73/142) of patients, respectively (see Figure 3).

Upadacitinib demonstrated significantly higher 3-month remission rate (*P* = .019) and 6-month response (*P* = .010) and remission rates (*P* = .001) compared to tofacitinib.

### Multivariable analysis

In multivariable logistic regression models including; gender, year of diagnosis, prior biologic count, smoking status and disease extent, adjusted odds ratios (aOR) for each JAKi were constructed for response and remission at 3 and 6 months using tofacitinib as the reference category. For response at 3 months, upadacitinib had an aOR of 2.72 (95% CI, 1.14-6.50, *P* = .025), filgotinib had an aOR of 1.53 (0.62-3.73, *P* = .354), with tofacitinib as the reference category. For remission at 3 months, upadacitinib had an aOR of 2.65 (1.34-5.23, *P* = .005) and filgotinib had an aOR of 2.36 (1.09-5.12, *P* = .029). For response at 6 months, upadacitinib had an aOR of 2.26 (1.10-4.62, *P* = .026) and filgotinib had an aOR of 1.69 (0.76-3.78, *P* = .198). For remission at 6 months, upadacitinib had an aOR of 2.86 (1.46-5.62, *P* = .002) and filgotinib had an aOR of 2.28 (1.05-4.95, *P* = .038). Full regression models can be found in Appendix Tables S2-S5.

**Figure 4:**
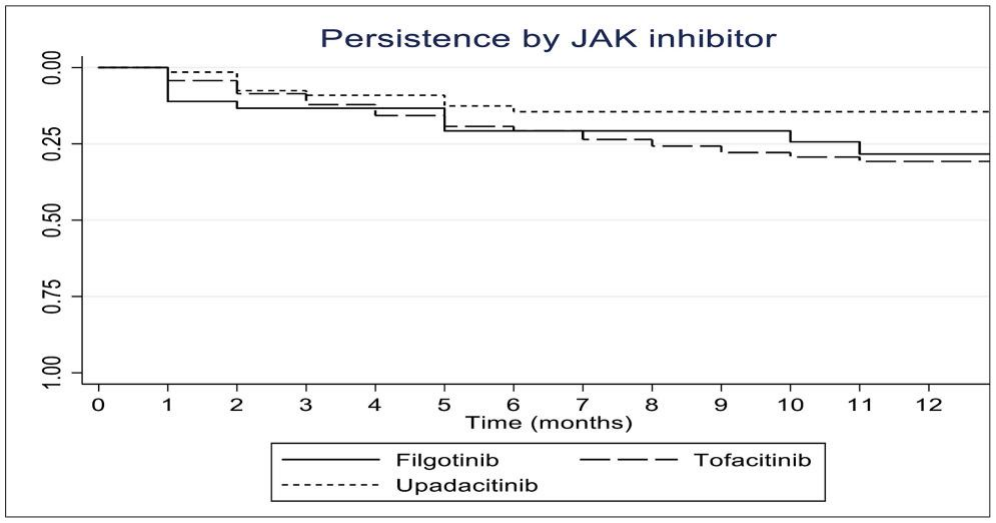
Persistence of treatment based on JAK inhibitor up to 12 months.

In the bio-exposed cohorts (*n* = 217), upadacitinib demonstrated greater 6-month combined remission rates (71%) compared to filgotinib (66%; *P*=NS) and tofacitinib (52%; *P* = .022) (Figure 4).

In bio-naïve cohorts (*n* = 53), upadacitinib demonstrated greater 3-month remission rates (93%) to filgotinib (79%; *P* = NS) and tofacitinib (50%, *P* = .009). Remission rates at 6 months for the bio-naïve cohorts also demonstrated upadacitinib to be superior in achieving greater remission rates (93%) compared to filgotinib (63%; *P* = .049) and tofacitinib (50%; *P* = .009) (Figure 4).

### Steroid-free remission

For all patients on JAKi, 89% on upadacitinib, 82% on filgotinib and 87% on tofacitinib were not taking corticosteroids at 3-month follow-up. At 6-month follow-up, 96% on upadacitinib, 100% on filgotinib and 92% on tofacitinib were corticosteroid-free.

Out of the 159 patients that were in disease remission at 3 months, 150 (94%) were not on corticosteroids (upadacitinib, 98%; filgotoinib, 94%; and tofacitinib, 92%). At 6 months, 98% (150/153) were in corticosteroid-free remission (upadacitinib, 98%; filgotinib, 100%; tofacitinib, 97%).

### Adverse events

A total of 3 patients reported episodes of herpes zoster: 2 on upadacitinib and 1 on tofacitinib. One patient developed a venous thromboembolic event (VTE) while on upadacitinib. This patient developed a VTE and underwent an emergency colectomy within 2 months of initiating upadacitinib.

A single major adverse cardiovascular event was noted in a patient using tofacitinib. This patient was over 60 years old, an ex-smoker and developed a transient ischemic attack (TIA).

A total of five cancers were diagnosed in four patients (2.7%) on tofacitinib and one (1.4%) patient on upadacitinib. The cancers diagnosed while on tofacitinib were basal cell carcinoma, colorectal cancer (with concomitant diagnosis of primary sclerosing cholangitis), melanoma, and unknown. The patient on upadacitinib was diagnosed with colorectal cancer. The range of exposure was 10-37 months after drug initiation with range between 4 and 34 years following UC diagnosis. One patient was an ex-smoker with the others having never smoked. All patients were over the age of 45 years and two of them over the age of 60 years. One patient was exposed to two previous biologics, and the other four patients were exposed to three or more biologics prior to JAKi initiation and cancer diagnosis.

Blood test abnormalities were observed in 58 patients, of whom the majority were lipid profile abnormalities; 18 (26%), 18 (12%). and 8 (17%) of patients on upadacitinib, tofacitinib, and filgotinib, respectively. A full breakdown of adverse events can be viewed in Appendix Table S6.

Hospital admissions (for any reason) were observed in 71 patients (26%) using JAKi, of whom 62 (87%) had prior biologic exposure. The incidence rates of hospital admission on upadacitinib, filgotinib, and tofacitinib were 10.2, 21.9, and 14.1 per 1000 patient-months at risk. Among admitted patients using upadacitinib, filgotinib, and tofacitinib—1/12 (8%), 3/11 (27%), and 5/48 (10%), respectively, were bio-naïve. Hospital admissions were linked to UC flare or drug side effects in almost all cases.

### Colectomy rates

A total of 26 patients (10%) had a colectomy while they were on their JAKi. The incidence rates of colectomy on upadacitinib, filgotinib, and tofacitinib were 6.7, 10.0, and 5.0 per 1000 patient-months at risk. In this group, 86% of those with endoscopic investigation had MES 3 with a median FCP of 1754 at baseline. Fifty-eight percent of patients had used two or more biologics prior to initiation on JAKi, and only one patient (on filgotinib) was bio-naïve prior to undergoing colectomy.

## Discussion

At the time of writing, this is the second retrospective study to compare efficacy between all three JAKi drugs with some key outcomes. First, our analysis demonstrated that upadacitinib consistently outperformed tofacitinib in each of the clinical, biochemical and endoscopic outcomes at both 3 and 6 months with statistically significantly greater efficacy in the 6-month response and remission rates. In multivariable logistic regression, both upadacitinib and filgotinib were more likely to achieve 3- and 6-month remission in patients with UC compared to tofacitinib. Upadacitinib also had the highest drug persistence at 12 months and was superior in both the bio-naïve and bio-exposed cohort. These findings are consistent with the recently published study by Akiyama et al. who also found upadacitinib to have higher efficacy and lower risk of discontinuation than patients treated with other JAKi.^9^

In this study, upadacitinib was the superior performing agent for the induction of clinical remission. This is in keeping with a recent systematic review and meta-analysis comparing the relative efficacy and safety of biologics and small molecules.^10^ To harness this real-world data, we used a combined variable analysis to offset the inconsistency in treatment assessment tools, which is often unavoidable in retrospective multicentered studies. Univariable analysis utilizing the combined variables of clinical indices, FCP, and endoscopic scoring demonstrated statistically significant greater efficacy of Upadacitinib compared to tofacitinib, and numerically greater efficacy of filgotinib compared to tofacitinib.

There were a significant number of adverse events within the JAK cohort. There was a VTE reported with upadacitinib, but because patients underwent a colectomy within 2 months of drug initiation, it is unclear whether it was the drug or disease severity that led to this adverse event.

There were also five cancers reported (2% of the total cohort), of which the majority of patients were older and more bio-exposed, intimating their disease was not well-controlled. It is well documented that chronic inflammatory conditions, such as IBD, with persistent inflammation foster a pro-tumorigenic environment, which increases the risk of malignancies.^11^ However, our reported findings are lower than the renowned 2022 ORAL Surveillance study demonstrating tofacitinib to have a cancer incidence of 4.2% in patients with rheumatoid arthritis.^12^ This was more commonly seen in patients older than 50 years with at least one additional cardiovascular risk factor. Despite this study, the increased cancer risk with tofacitinib has not been conclusively established in IBD patients, particularly in those without significant baseline risk factors, such as age, smoking history, or prior malignancy.

Even with multiple therapeutic options, patients with UC are recorded to have an estimated 10% risk of colectomy within 10 years^13^ and a 20% risk of UC-related hospitalization within 5 years of diagnosis.^14^ Recent evidence^15–17^ suggests that tofacitinib may provide a protective effect against colectomy (hazard ratio 0.28, 95% CI, 0.10-0.81, *P* = .018).^18^ While our findings were similar to the literature in that a total of 10% of the patient cohort underwent a colectomy, there was little variation in the incidence rate of colectomy in the tofacitinib group versus the upadacitinib and filgotinib groups. This is in contrast to Kochhar et al. who found that upadacitinib had a lower risk for colectomy compared to tofacitinib.^19^ However, we did find that our patients had severe disease activity and were more likely to be bio-exposed, with only 4% undergoing colectomies bio-naïve.

There have been limited studies comparing JAKi, and all of them have been retrospective, with only one study comparing all three JAKi.^9^^,^^19–21^ The results have been similar to our study with upadacitinib proving to be the superior drug, not only with greater clinical remission rates but also in reducing hospitalization and colectomy rates. These studies used clinical metrics as analysis outcomes, but our study had the added advantage of including endoscopic analysis. Although our numbers were small, it would be unexpected for patients to have endoscopic assessment so soon after treatment initiation. From the small numbers, however, our results do demonstrate that upadacitinib had numerically higher endoscopic improvement compared to filgotinib and tofacitinib at both 3 and 6 months.

A recent meta-analysis of real-world evidence for tofacitinib in UC from 2021 offers results compatible with our own.^22^ They report a pooled induction cohort at 8-14 weeks post-initiation with a 95% confidence interval for clinical remission of 26%-45% compared to 52% at the same time point in this study. However, among 342 pooled patients, 109 (32%) had exposure to ≥3 prior biologics, compared to 7% in the cohort reported in the present study. The cohort reported in this manuscript were initiated on a JAKi more recently and therefore likely represent more up-to-date approaches for sequencing advanced therapies for UC. It is therefore not surprising that JAKi treatment outcomes observed in the present study may appear favorable compared to other observations of real-world data.

The main limitation to this study is the retrospective aspect with inconsistencies in measuring treatment response tools. However, it is unlikely we will ever have prospective head-to-head studies between biologics or small molecules and so real-world studies, within its confines, are needed to compare drug efficacy. As stated above, we attempted to counteract this limitation by demonstrating both independent and combined variable outcomes. Our results (also shown in Figure S1) demonstrates that these measures correlate well, supporting the validity of a combined outcome measure. Furthermore, although patients were not randomized between the different JAKi medications, analysis of the baseline demographics and disease variables did not detect any significant variation. The exception to this is that a higher proportion of patients receiving filgotinib were bio-naive, which might have led to an over-estimation of the efficacy of filgotinib compared to tofacitinib and upadacitinib. However, considering that upadacitinib appeared to be the most efficacious JAKi in this study, this has not unduly influenced the interpretation of our findings.

Other limitations include lower recruitment numbers for filgotinib and upadacitinib than tofacitinib, which is reflective of the length of time that these medications have been licensed in the UK. However, this may also demonstrate the real-world use of these medications capturing the true clinical use and experience of these medications. Finally, the number of patients who underwent endoscopic assessment were small. While upadacitinib demonstrated numerically higher endoscopic response rates compared to the other JAKi, these results were not statistically significant and thus, should be interpreted with caution.

It should be noted that a power calculation was not undertaken in this retrospective study. However, considering the level of statistical significance and consistency of findings at 3 and 6 months for both clinical response and remission, this study still strongly supports the difference in outcomes observed. Furthermore, drug dosing was generally homogenous, with an almost unanimous use of 30 mg maintenance dose Upadacitinib and 5 mg tofacitinib.

In summary, in this real-world study upadacitinib and filgotinib were observed to be more likely to achieve 6 months clinical response and remission rates compared to tofacitinib in multivariable analysis. Upadacitinib was found to be similarly effective in both the bio-naïve and bio-exposed cohort. Further larger, prospective studies are now needed to validate this data.

## Supplementary Material

otag045_Supplementary_Data

## Data Availability

The data underlying this article will be shared on reasonable request to the corresponding author.
