# Supplementary material for: A multicentered retrospective cohort study comparing JAK inhibitor therapies in moderate-to-severe ulcerative colitis
Source: Crohns Colitis 360. 2026 May 26;8(2):otag045. doi: 10.1093/crocol/otag045 (PMC13255995; doi:10.1093/crocol/otag045)
Supplement: otag045_Supplementary_Data [file otag045_supplementary_data.docx]

**SUPPLEMENTARY FILE**

**A multi-centred retrospective cohort study comparing JAK inhibitor therapies in moderate-to-severe ulcerative colitis**

**Appendix table 1: Remission rates at 3- and 6-months by assessment modality and JAK inhibitors**

|  | **3-months** | | | **6-months** | | |
| --- | --- | --- | --- | --- | --- | --- |
|  | **Clinical** | **Biochemical** | **Endoscopic** | **Clinical** | **Biochemical** | **Endoscopic** |
| **Upadacitinib** | 74%  (34/46) | 69%  (27/39) | 83%  (5/6) | 91%  (21/23) | 71%  (17/24) | 80%  (4/5) |
| **Filgotinib** | 84%  (26/31) | 71%  (17/24) | 67%  (2/3) | 78%  (14/18) | 67%  (10/15) | 50%  (1/2) |
| **Tofacitinib** | 66%  (73/111) | 51%  (41/80) | 25%  (3/12) | 73%  (59/81) | 51%  (37/68) | 44%  (4/9) |

**Appendix table 2: Logistic regression model of factors associated with response at 3 months**

| **Variable** |  | **Odds ratio** | **95% CI** | **p value** |
| --- | --- | --- | --- | --- |
| **JAK inhibitor** | **Tofacitinib** | Ref cat |  |  |
|  | **Filgotinib** | 1.53 | 0.62 - 3.73 | 0.354 |
|  | **Upadacitinib** | 2.72 | 1.14 - 6.50 | 0.025 |
| **Disease extent** | **E1** | Ref cat |  |  |
|  | **E2** | 0.38 | 0.08 - 1.85 | 0.231 |
|  | **E3** | 0.57 | 0.12 - 2.85 | 0.496 |
|  | **Missing** | 0.07 | 0.01 - 0.77 | 0.030 |
| **Smoking status** | **Current** | Ref cat |  |  |
|  | **Never** | 0.97 | 0.07 – 14.1 | 0.983 |
|  | **Previous** | 1.24 | 0.08 - 20.3 | 0.881 |
|  | **Unknown** | 1.09 | 0.06 – 19.2 | 0.952 |
| **Prior biologic count** | **Bio-naïve** | Ref cat |  |  |
|  | **1** | 0.66 | 0.25 – 1.72 | 0.396 |
|  | **2** | 0.36 | 0.14 – 0.97 | 0.043 |
|  | **3+** | 1.06 | 0.18 – 6.06 | 0.950 |
| **Year of diagnosis** | **2021-2024** | Ref cat |  |  |
|  | **2018-2020** | 1.70 | 0.69 – 4.22 | 0.252 |
|  | **2015-2017** | 2.20 | 0.76 – 6.37 | 0.145 |
|  | **2014 or earlier** | 1.95 | 0.80 – 4.73 | 0.139 |
| **Female Gender** |  | 1.21 | 0.633 – 2.29 | 0.568 |

**Appendix table 3: Logistic regression model of factors associated with remission at 3 months**

| **Variable** |  | **Odds ratio** | **95% CI** | **p value** |
| --- | --- | --- | --- | --- |
| **JAK inhibitor** | **Tofacitinib** | Ref cat |  |  |
|  | **Filgotinib** | 2.36 | 1.09 – 5.12 | 0.029 |
|  | **Upadacitinib** | 2.65 | 1.34 – 5.23 | 0.005 |
| **Disease extent** | **E1** | Ref cat |  |  |
|  | **E2** | 0.54 | 0.17 – 1.70 | 0.295 |
|  | **E3** | 0.57 | 0.18 – 1.81 | 0.344 |
|  | **Missing** | 0.10 | 0.01 – 0.83 | 0.033 |
| **Smoking status** | **Current** | Ref cat |  |  |
|  | **Never** | 0.43 | 0.04 – 4.80 | 0.205 |
|  | **Previous** | 0.62 | 0.05 - 7.65 | 0.084 |
|  | **Unknown** | 0.40 | 0.03 – 5.06 | 0.267 |
| **Prior biologic count** | **Bio-naïve** | Ref cat |  |  |
|  | **1** | 0.61 | 0.28 - 1.31 | 0.205 |
|  | **2** | 0.49 | 0.21 – 1.10 | 0.084 |
|  | **3+** | 0.53 | 0.17 – 1.63 | 0.267 |
| **Year of diagnosis** | **2021-2024** | Ref cat |  |  |
|  | **2018-2020** | 1.08 | 0.50 - 2.32 | 0.848 |
|  | **2015-2017** | 2.67 | 1.06 – 6.74 | 0.037 |
|  | **2014 or earlier** | 1.13 | 0.54 – 2.39 | 0.739 |
| **Female Gender** |  | 1.47 | 0.85 – 2.54 | 0.167 |

**Appendix table 4: Logistic regression model of factors associated with response at 6 months**

| **Variable** |  | **Odds ratio** | **95% CI** | **p value** |
| --- | --- | --- | --- | --- |
| **JAK inhibitor** | **Tofacitinib** | Ref cat |  |  |
|  | **Filgotinib** | 1.71 | 0.75 – 3.88 | 0.200 |
|  | **Upadacitinib** | 2.75 | 1.26 – 6.01 | 0.011 |
| **Disease extent** | **E1** | Ref cat |  |  |
|  | **E2** | 0.22 | 0.04 – 1.13 | 0.070 |
|  | **E3** | 0.18 | 0.04 – 0.96 | 0.045 |
|  | **Missing** | 0.06 | 0.01 – 0.60 | 0.017 |
| **Smoking status** | **Current** | Ref cat |  |  |
|  | **Never** | 2.00 | 0.25 – 16.1 | 0.514 |
|  | **Previous** | 4.23 | 0.45 – 40.1 | 0.209 |
|  | **Unknown** | 5.17 | 0.48 – 56.0 | 0.177 |
| **Prior biologic count** | **Bio-naïve** | Ref cat |  |  |
|  | **1** | 1.20 | 0.54 – 2.70 | 0.654 |
|  | **2** | 0.66 | 0.28 – 1.53 | 0.330 |
|  | **3+** | 2.77 | 0.52 – 14.7 | 0.231 |
| **Year of diagnosis** | **2021-2024** | Ref cat |  |  |
|  | **2018-2020** | 0.82 | 0.36 – 1.84 | 0.630 |
|  | **2015-2017** | 1.52 | 0.57 – 4.03 | 0.398 |
|  | **2014 or earlier** | 1.45 | 0.63 – 3.34 | 0.380 |
| **Female Gender** |  | 1.52 | 0.84 – 2.76 | 0.169 |

**Appendix table 5: Logistic regression model of factors associated with remission at 6 months**

| **Variable** |  | **Odds ratio** | **95% CI** | **p value** |
| --- | --- | --- | --- | --- |
| **JAK inhibitor** | **Tofacitinib** | Ref cat |  |  |
|  | **Filgotinib** | 2.63 | 1.18 – 5.92 | 0.019 |
|  | **Upadacitinib** | 4.08 | 1.92 – 8.68 | <0.001 |
| **Disease extent** | **E1** | Ref cat |  |  |
|  | **E2** | 0.36 | 0.10 – 1.29 | 0.117 |
|  | **E3** | 0.35 | 0.10 – 1.24 | 0.104 |
|  | **Missing** | 0.06 | 0.01 – 0.51 | 0.010 |
| **Smoking status** | **Current** | Ref cat |  |  |
|  | **Never** | 1.60 | 0.20 – 12.4 | 0.655 |
|  | **Previous** | 3.45 | 0.39 – 30.7 | 0.267 |
|  | **Unknown** | 2.37 | 0.24 – 23.5 | 0.461 |
| **Prior biologic count** | **Bio-naïve** | Ref cat |  |  |
|  | **1** | 1.07 | 0.49 – 2.34 | 0.866 |
|  | **2** | 0.60 | 0.26 – 1.37 | 0.225 |
|  | **3+** | 0.82 | 0.25 – 2.71 | 0.747 |
| **Year of diagnosis** | **2021-2024** | Ref cat |  |  |
|  | **2018-2020** | 1.23 | 0.55 – 2.75 | 0.614 |
|  | **2015-2017** | 2.42 | 0.93 – 6.34 | 0.072 |
|  | **2014 or earlier** | 1.43 | 0.65 – 3.19 | 0.374 |
| **Female Gender** |  | 1.22 | 0.69 - 2.14 | 0.500 |

**Supplementary Figure 1: Proportion of patients meeting remission criteria by clinical score, faecal calprotectin biomarker and endoscopic assessment at baseline, 3 months and 6 months for all JAK inhibitors and each drug individually.**

**
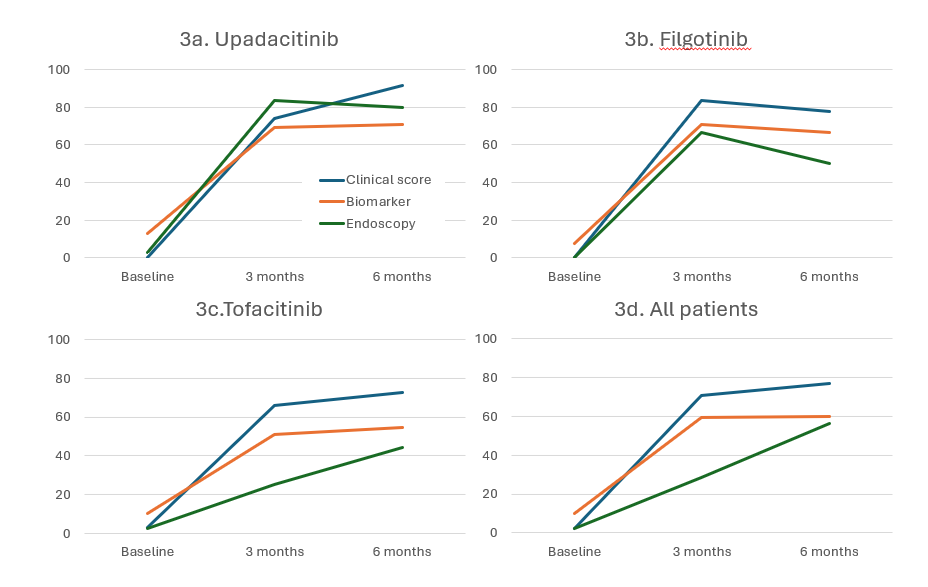
**

***Appendix Table 6:*** *Adverse events in JAK inhibitors*

| **Adverse events** | | **Tofacitinib**  **N=149** | **Filgotinib**  **N=51** | **Upadacitinib**  **N=70** |
| --- | --- | --- | --- | --- |
| Infections | |  |  |  |
|  | Herpes Zoster | 1 (0.6%) | 0 | 2 (2.9%) |
| Venous thromboembolism | | 1 (0.6%) | 0 | 0 |
| Major adverse cardiovascular event | | 1 (0.6%) | 0 | 0 |
| Malignancy | | 2 (1.3%) | 0 | 0 |
| Blood test abnormalities | | 18 (12%) | 8 (16%) | 18 (26%) |
| Hospitalisation | | 48 (32%) | 11 (22%) | 12 (17%) |
| Colectomy | | 17 (65%) | 5 (10%) | 4 (15%) |
